# Late-onset group B streptococcal septic arthritis in an afebrile neonate: a case report

**DOI:** 10.3389/fped.2026.1878171

**Published:** 2026-08-19

**Authors:** Asuman Akar

**Affiliations:** Department of Pediatrics, Division of Pediatric Infectious Diseases, Dicle University Faculty of Medicine, Diyarbakır, Türkiye

**Keywords:** case report, group B Streptococcus, late-onset GBS disease, neonatal septic arthritis, urine PCR

## Abstract

The majority of neonatal group B streptococcal (GBS) infections present as sepsis or meningitis, while osteoarticular involvement is rare during the neonatal period. We report a case of culture-confirmed late-onset GBS septic arthritis in a 26-day-old male neonate presenting with progressive swelling, erythema, and irritability of the left knee without fever. Surgical irrigation and debridement revealed purulent arthritis. Because maternal prenatal records, maternal GBS screening status, and maternal infectious disease history were unavailable, a catheterized urine polymerase chain reaction (PCR) panel for sexually transmitted infections and genital tract-associated pathogens was obtained during the evaluation of a possible vertically or perinatally transmitted infection. The panel was positive for GBS on hospital day 0, approximately 72 h before joint tissue culture confirmation. Joint tissue culture subsequently yielded GBS, while blood and cerebrospinal fluid cultures remained sterile. The patient was treated successfully with intravenous ampicillin after initial empiric therapy with ampicillin and gentamicin. Clinical recovery and ultrasonographic resolution of joint effusion were achieved, and no clinical sequelae were detected at the 3-month post-discharge follow-up. This case emphasizes that afebrile neonates with progressive monoarticular swelling should be evaluated for septic arthritis and that definitive diagnosis relies on microbiological confirmation from a sterile-site specimen. The urine PCR finding, in this case, is reported as a hypothesis-generating observation in a neonate with unavailable maternal screening data; however, its biological significance remains uncertain and should not be interpreted as validation of urine PCR for invasive neonatal GBS disease or septic arthritis*.*

## Introduction

Group B Streptococcus (GBS) remains a leading cause of neonatal invasive disease worldwide (1). The majority of neonatal GBS infections present as sepsis or meningitis, while osteoarticular involvement is uncommon and has been reported only rarely during late-onset disease (2–5). We report a case of late-onset GBS septic arthritis in an afebrile neonate. A catheterized urine polymerase chain reaction (PCR) panel for sexually transmitted infections and genital tract-associated pathogens was positive for GBS on hospital day 0, approximately 72 h before joint tissue culture confirmation; this finding is discussed as a hypothesis-generating observation rather than as a validated diagnostic method.

## Case presentation

The male neonate was born at 39 weeks’ gestation via vaginal delivery with a birth weight of 3,400 g. Maternal prenatal records, GBS screening results, and infectious disease history were unavailable. At 10 days of life, the infant began to be cared for by another legal caregiver. Left knee swelling was first noticed on day 5 of life. Because there was no fever, medical attention was not sought initially, despite progressive irritability during diaper changes.

On day 26 of life, the infant presented with worsening swelling and erythema of the left knee, accompanied by marked irritability. On examination, body weight was 4,500 g. Temperature was 36.7 °C, heart rate 120/min, respiratory rate 35/min, blood pressure 86/55 mmHg, and oxygen saturation 100% on room air. The anterior fontanelle was normal. Cardiopulmonary and abdominal examinations were unremarkable. A local examination of the left knee showed swelling, erythema, warmth, tenderness, and painful limitation of passive movement. There was no skin wound, signs of trauma, rash, hepatosplenomegaly, or abnormalities in other joints. Distal pulses and perfusion were preserved. Laboratory studies showed a white blood cell count of 12,700/µL with 14% neutrophils and a C-reactive protein (CRP) level of 142 mg/L. Orthopedic consultation was obtained after imaging evaluation.

### Diagnostic assessment and differential diagnosis

The initial diagnostic evaluation included blood and urine cultures. A lumbar puncture was performed because invasive neonatal infection was considered in the differential diagnosis and central nervous system involvement needed to be excluded. Cerebrospinal fluid (CSF) was sent for culture and a meningitis PCR panel. The differential diagnosis included septic arthritis, osteomyelitis, cellulitis, traumatic hemarthrosis, non-accidental injury, and vertically or perinatally transmitted infections, including gonococcal or syphilitic disease. Septic arthritis was considered the leading diagnosis given progressive monoarticular swelling, irritability with movement, elevated CRP, and imaging evidence of joint effusion.

During surgery, purulent material was identified in the joint space. Aspiration, irrigation, and debridement were performed, and tissue samples were obtained for microbiological analysis. Because maternal prenatal records, GBS screening status, and infectious disease history were unavailable, serologic screening for HIV, hepatitis B, hepatitis C, and syphilis was performed; all the results were negative. In addition, a urine specimen was obtained aseptically via sterile catheterization and tested using the Bio-Speedy® Sexually Transmitted Infections RT-qPCR Panel, also known locally as the Bio-Speedy® CYBH Panel (Bioeksen AR-GE Teknolojileri A.Ş., Istanbul, Türkiye). This qualitative multiplex RT-qPCR panel includes GBS among its bacterial targets. The urine PCR result was positive for GBS on hospital day 0, approximately 72 h before joint tissue culture confirmation on hospital day 3. The cycle threshold (Ct) values were not reported by the laboratory. Repeat urine PCR testing was not performed. Because this assay has not been validated for diagnosing invasive neonatal GBS disease or septic arthritis using urine specimens, the result was only interpreted as an ancillary microbiological finding, not as confirmatory evidence of invasive disease.

Empiric intravenous antimicrobial therapy was initiated postoperatively with ampicillin at 100 mg/kg/dose every 8 h and gentamicin at 4 mg/kg/dose every 24 h. On hospital day 3, joint tissue culture yielded GBS, while blood and CSF cultures remained sterile and no pathogen was detected on the CSF meningitis PCR panel. Antimicrobial susceptibility testing of the joint tissue isolate was performed using the BD Phoenix™ system (NJ, USA) and interpreted according to European Committee on Antimicrobial Susceptibility Testing criteria; the isolate was susceptible to ampicillin, with a minimum inhibitory concentration (MIC) of 0.12 µg/mL. Because our institution applies selective/cascade antimicrobial susceptibility reporting as part of antimicrobial stewardship, only the clinically relevant ampicillin susceptibility result was released in the routine laboratory report. No additional antimicrobial MIC values were reported. Based on culture confirmation, ampicillin susceptibility, persistently negative blood and CSF cultures, and clinical and laboratory improvement, gentamicin was discontinued on treatment day 7, and targeted intravenous ampicillin therapy was continued for a total of 30 days. No treatment-related adverse effects were observed. Follow-up ultrasonography demonstrated complete resolution of the joint effusion. At the 3-month post-discharge follow-up, the infant was re-evaluated in the pediatric infectious diseases, orthopedics, and physical medicine and rehabilitation clinics. No swelling, erythema, pain, limitation of passive or active movement, limb-use asymmetry, or clinically evident orthopedic sequelae was detected. Magnetic resonance imaging (MRI) was planned for further assessment but could not be completed during the documented follow-up period. Therefore, long-term follow-up by pediatric infectious diseases and orthopedics is ongoing, and further imaging will be guided by clinical findings (Table 1).

**Table 1:** Timeline of clinical presentation, diagnostic work-up, treatment, and follow-up.

Time point	Event	Diagnostic/therapeutic actions and outcome
Day 0 of life (birth)	Term male neonate born by vaginal delivery, birth weight 3,400 g	Routine neonatal care; stable after birth
Day 5 of life	Left knee swelling first noticed	No medical evaluation was sought because the infant was afebrile; symptoms progressed
Day 26 of life (hospital day 0)	Hospital presentation with worsening left knee swelling, erythema, and irritability	Blood and urine cultures, CSF evaluation, meningitis PCR panel, imaging, and orthopedic consultation were performed. Surgical aspiration, irrigation, and debridement were performed; tissue was sent for culture. Empiric intravenous ampicillin and gentamicin were started. Catheterized urine PCR panel was positive for GBS
Day 29 of life (hospital day 3)	Joint tissue culture yielded GBS	Blood, urine, and CSF cultures remained sterile, and the CSF meningitis PCR panel was negative. The isolate was susceptible to ampicillin, with MIC 0.12 µg/mL
Day 33 of life (hospital day 7)	Clinical and laboratory improvement	Blood and CSF cultures remained negative. Gentamicin was discontinued, and targeted intravenous ampicillin was continued
Day 56 of life (hospital day 30)	Completion of intravenous ampicillin therapy	No treatment-related adverse effects were observed. The infant was discharged with outpatient follow-up
Day 146 of life approximately (3-month follow-up)	Pediatric infectious diseases, orthopedics, and physical medicine and rehabilitation follow-up	Ultrasonography showed resolution of joint effusion. No clinically evident orthopedic sequelae were detected

## Discussion

GBS possesses multiple virulence factors, including capsular polysaccharides and surface proteins, that contribute to invasive neonatal disease (5). Universal maternal screening and intrapartum antibiotic prophylaxis have significantly reduced the incidence of early-onset GBS infection (6). Nevertheless, inadequate prenatal follow-up continues to undermine the effectiveness of preventive strategies in vulnerable populations.

Late-onset GBS disease is defined as infection occurring from day 7 through to day 89 of life, and transmission routes include maternal, household, breast milk, nosocomial, and community sources (7). Invasive disease can cause sepsis, meningitis, and long-term disability, and global burden estimates remain high (8, 9).

Osteoarticular involvement is an uncommon manifestation of neonatal GBS disease rather than a typical presentation. GBS septic arthritis in neonates may present insidiously without fever or systemic instability, potentially delaying diagnosis (3, 4, 10). Reported neonatal GBS joint infections include monoarthritis, polyarthritis, and osteomyelitis with septic arthritis, but these are described mainly in isolated case reports or small series (11). Prompt diagnosis matters because neonatal septic arthritis can rapidly damage cartilage and growth plates when treatment is delayed (4). Current pediatric guidelines emphasize the importance of joint fluid analysis, tissue culture, and blood cultures for osteoarticular infections (12). Imaging is also important, and prior reports of neonatal GBS arthritis note that ultrasonography may miss early effusions, whereas MRI can detect subtle joint inflammation in uncertain cases (3). This aligns with surveillance definitions of invasive GBS disease, which rely on isolation from a normally sterile site (13).

The biological meaning of urinary GBS DNA detection in this neonate remains uncertain. The broader PCR literature mainly addresses maternal GBS colonization screening rather than diagnosis of invasive neonatal septic arthritis from urine (17, 18). In maternal screening studies, qPCR may detect colonization more frequently than culture, but culture remains the reference method in many protocols. In the present case, the catheterized urine PCR panel was positive for GBS on hospital day 0, approximately 72 h before sterile-site culture confirmation. However, urinary detection of GBS DNA does not prove invasive osteoarticular infection. It cannot be determined from this single case whether urine PCR positivity reflected genitourinary colonization, transient urinary shedding, urinary tract involvement, contamination despite catheter-based collection, or a sign related to systemic dissemination. The negative urine culture and the absence of urinary tract-specific clinical findings do not support defining the urinary tract as the primary infectious focus. Conversely, the negative blood culture does not exclude transient hematogenous spread, particularly because blood culture sensitivity may be affected by low-level or intermittent bacteremia. Therefore, the clinically confirmed invasive focus in this case was the joint, based on sterile-site culture, whereas the urine PCR result should be interpreted only as a hypothesis-generating microbiological observation. Published data on urine-based molecular detection of GBS in neonates are extremely limited. Cezarino et al. evaluated nested PCR using residual urine and other specimens in neonates with suspected or confirmed GBS sepsis; however, that study involved neonatal sepsis, used a nested PCR method rather than a commercial sexually transmitted infection (STI) multiplex RT-qPCR panel, and did not address osteoarticular infection or septic arthritis (14). We did not identify previous reports in which a catheterized urine STI multiplex PCR panel was positive for GBS in a neonate with culture-confirmed septic arthritis. Therefore, the present observation should not be generalized to clinical practice and requires prospective evaluation before any diagnostic role can be proposed.

Because late-onset GBS disease may be associated with meningitis and central nervous system involvement, CSF evaluation should be considered in neonates with suspected invasive GBS infection (15). In our patient, CSF culture remained sterile and no pathogen was detected on the CSF meningitis PCR panel. Management of neonatal septic arthritis requires prompt source control and pathogen-directed antimicrobial therapy. Penicillin and ampicillin remain first-line agents for invasive GBS disease in reviews and guideline-based sources (4, 9, 16). Surgical drainage is often part of successful management in neonatal septic arthritis and improves source control and microbiological yield (11). Our patient underwent surgical irrigation and debridement and responded favorably to 30 days of targeted intravenous ampicillin therapy.

## Strengths and limitations

The main strength of this report is the culture-confirmed diagnosis of neonatal GBS septic arthritis from a sterile-site surgical specimen, together with detailed microbiological, therapeutic, and follow-up information. The main limitations are the single-patient nature of the observation, the lack of Ct values or repeat urine PCR testing, the uncertain biological significance of urinary GBS DNA detection, and the absence of serotyping and molecular typing of the bacterial isolate. Because the documented follow-up period was limited to 3 months after discharge and MRI could not be completed, late growth disturbance or subtle osteoarticular sequelae cannot be definitively excluded.

## Conclusion

Neonatal GBS septic arthritis is rare and may present without fever or systemic instability. In afebrile neonates with progressive monoarticular swelling or irritability during movement, septic arthritis should be considered and sterile-site microbiological sampling should be obtained promptly. In this case, the diagnosis was confirmed by growth of GBS from joint tissue culture. The positive urine PCR result was an ancillary observation with uncertain biological significance and should not be interpreted as validation of urine PCR for diagnosing invasive neonatal GBS disease or septic arthritis. This urine PCR finding should therefore be considered hypothesis-generating only, and further validation studies are required before any clinical role for urine-based molecular testing in invasive neonatal GBS disease can be proposed.

## Patient perspective

Because the patient was a neonate, a direct patient perspective could not be obtained. The infant's legal guardian/caregiver was informed about the diagnosis, surgical intervention, antimicrobial treatment, follow-up plan, and clinical outcome.

## Data Availability

The raw data supporting the conclusions of this article will be made available by the authors, without undue reservation.
